# TcEVdb: a database for T-cell-derived small extracellular vesicles from single-cell transcriptomes

**DOI:** 10.1093/database/baaf012

**Published:** 2025-02-28

**Authors:** Tao Luo, Wen-Kang Shen, Chu-Yu Zhang, Dan-Dan Song, Xiu-Qing Zhang, An-Yuan Guo, Qian Lei

**Affiliations:** College of Life Sciences, University of Chinese Academy of Sciences, Beijing 100049, China; Department of thoracic surgery, West China Biomedical Big Data Center, West China Hospital, Sichuan University, #37 Guoxue Alley, Wuhou District, Chengdu 610041, China; Department of thoracic surgery, West China Biomedical Big Data Center, West China Hospital, Sichuan University, #37 Guoxue Alley, Wuhou District, Chengdu 610041, China; Hubei Bioinformatics & Molecular Imaging Key Laboratory, College of Life Science and Technology, Huazhong University of Science and Technology, 1037 Luoyu Road, Hongshan District, Wuhan 430074, China; Department of thoracic surgery, West China Biomedical Big Data Center, West China Hospital, Sichuan University, #37 Guoxue Alley, Wuhou District, Chengdu 610041, China; Hubei Bioinformatics & Molecular Imaging Key Laboratory, College of Life Science and Technology, Huazhong University of Science and Technology, 1037 Luoyu Road, Hongshan District, Wuhan 430074, China; Department of thoracic surgery, West China Biomedical Big Data Center, West China Hospital, Sichuan University, #37 Guoxue Alley, Wuhou District, Chengdu 610041, China; College of Life Sciences, University of Chinese Academy of Sciences, Beijing 100049, China; Department of thoracic surgery, West China Biomedical Big Data Center, West China Hospital, Sichuan University, #37 Guoxue Alley, Wuhou District, Chengdu 610041, China; Department of thoracic surgery, West China Biomedical Big Data Center, West China Hospital, Sichuan University, #37 Guoxue Alley, Wuhou District, Chengdu 610041, China

## Abstract

T-Cell-derived extracellular vesicles (TcEVs) play key roles in immune regulation and tumor microenvironment modulation. However, the heterogeneity of TcEV remains poorly understood due to technical limitations of EV analysis and the lack of comprehensive data. To address this, we constructed TcEVdb, a comprehensive database that explores the expression and cluster of TcEV by the SEVtras method from T-cell single-cell RNA sequencing data. TcEVdb contains 277 265 EV droplets from 51 T-cell types across 221 samples from 21 projects, covering 9 tissue sources and 23 disease conditions. The database provides two main functional modules. The Browse module enables users to investigate EV secretion activity indices across samples, visualize TcEV clusters, analyze differentially expressed genes (DEGs) and pathway enrichment in TcEV subpopulations, and compare TcEV transcriptomes with their cellular origins. The Search module allows users to query specific genes across all datasets and visualize their expression distribution. Furthermore, our analysis of TcEV in diffuse large B-cell lymphoma revealed increased EV secretion in CD4^+^ T exhausted cells compared to healthy controls. Subsequent analyses identified distinct droplet clusters with differential expression genes, including clusters enriched for genes associated with cell motility and mitochondrial function. Overall, TcEVdb serves as a comprehensive resource for exploring the transcriptome of TcEV, which will contribute to advancements in EV-based diagnostics and therapeutics across a wide range of diseases.

**Database URL**: https://guolab.wchscu.cn/TcEVdb

## Introduction

Extracellular vesicles (EVs) are lipid bilayer-enclosed vesicles secreted by nearly all cell types, carrying a diverse array of biological molecules, including proteins, lipids, and nucleic acids [1]. They act as intercellular messengers and play crucial roles in intercellular communication and various pathological processes [2, 3]. Specifically, T-cell-derived EV (TcEV) have emerged as critical mediators in immune regulation, modulating immune responses, shaping the tumor microenvironment, and influencing the pathogenesis of autoimmune diseases [4–6]. However, the high heterogeneity of the EV population presents a significant challenge in elucidating their specific functions [3, 7].

In the EV research field, one of the most attractive and challenging questions is to understand the heterogeneity and functional diversity of EVs [8]. Traditional bulk analysis techniques often obscure this complexity, potentially masking important subpopulations with distinct functional characteristics. Recent technological advancements have made remarkable progress in single-vesicle-level analysis, employing techniques such as nanoflow cytometry [9], total internal reflection fluorescence microscopy [10], microfluidic devices [11], and single-EV sequencing [12]. While these methods have provided valuable insights into EV heterogeneity, they fall short of enabling high-throughput analysis of EVs derived from a wide variety of cell types.

The advent of droplet-based single-cell RNA sequencing (scRNA-seq) has revolutionized the investigation of cellular heterogeneity at single-cell resolution. Recently, He *et al*. demonstrated that scRNA-seq data contain information about EVs, which are captured alongside individual cells in droplets. Based on this insight, they developed SEVtras, an innovative algorithm designed to identify EV-containing droplets in scRNA-seq data and estimate the EV secretion activity of individual cells [13]. SEVtras represents a significantly advancement in EV research by enabling the characterization and analyzing the expression of single or few EVs within scRNA-seq data. This approach facilitates high-throughput analysis of EV heterogeneity and secretion patterns, leveraging extensive existing scRNA-seq data without necessitating specialized EV isolation techniques.

Despite these advancements, a comprehensive resource for exploring TcEV across diverse tissues and pathological conditions remains unavailable. To address this gap, we present TcEVdb (a database for T-cell-derived small extracellular vesicles), a novel database that provides an unprecedented view of TcEV from scRNA-seq data. TcEVdb consists of 277 265 EV droplets from 21 projects containing 211 samples, encompassing 9 tissue sources and 23 different disease states and treatment conditions. By providing this comprehensive resource, TcEVdb will catalyze new discoveries in the field of TcEV and contribute to the advancement of EV-based diagnostics and therapeutics.

## Materials and Methods

### Data collection and preprocessing

We systematically collected T-cell scRNA-seq datasets from public repositories, including the National Center for Biotechnology Information Sequence Read Archive (SRA) (http://www.ncbi.nlm.nih.gov/sra) and the European Molecular Biology Laboratory - European Bioinformatics Institute BioStudies database [14], utilizing the keywords “T cell” and “scRNA-seq” to identify relevant datasets, and subsequently selecting only those with the organism specified as Homo sapiens. For each dataset, we documented relevant metadata, which included disease state, tissue source, sequencing platform information, and corresponding PubMed article references. Raw sequencing data in SRA format were downloaded using the sratoolkit (https://github.com/ncbi/sra-tools) prefetch command and subsequently converted to FASTQ format with the fastq-dump command. Quality control of the sequencing reads was conducted using fastp (v0.20.1) with default parameters [15, 16].

The quality-controlled reads were aligned to the built-in human reference genome (GRCh38) using Cell Ranger (v6.0.1). The resulting gene expression matrixes underwent further analysis using the Scanpy package [17] in Python3 (https://www.python.org/). Quality control was performed at the cellular level, retaining cells with gene detection counts ranging from 300 to 6500, total counts between 500 and 40 000, and mitochondrial gene expression below 15%. Gene-level filtering retained genes detected in more than three cells. Mitochondrial and ribosomal genes were excluded from further analysis.

To isolate T cells from the scRNA-seq data, we retained cells expressing CD3D or CD3G, while removing cells marked by B-cell (CD79A), epithelial cell (EPCAM), or fibroblast (PDGFR4) markers. Potential doublets were filtered out using Scrublet [18].

### Cell type annotation

Cell type annotation was performed using a two-step approach that combined automated and manual annotation. Firstly, we employed CellTypist [19] for the initial automated annotation, utilizing five built-in models (Immune_All_Low, COVID19_HumanChallenge_Blood, Healthy_COVID19_PBMC, Cells_Intestinal_Tract, and Cells_Lung_Airway). These models primarily focused on annotating major CD4^+^ T-cell subtypes, CD8^+^ T-cell subtypes, nonconventional T cells, and other cell types. For each cell, the most frequently predicted cell type across the five models was assigned as the initial classification, after which cells classified as “others” were excluded.

In the manual annotation step, we employed a marker-based annotation method based on the results of the automated annotation. To achieve a more granular annotation of T-cell subtypes and states, we collected a comprehensive list of marker genes from an extensive literature review (Supplementary Table S2). Leveraging the Scanpy [17] package, we implemented scRNA-seq data analysis steps. First, dimensionality reduction was performed using the Uniform Manifold Approximation and Projection (UMAP) algorithm (sc.tl.umap). Subsequently, unsupervised clustering was conducted using the Leiden algorithm (sc.tl.leiden) with a resolution set to 2.0. Marker genes for each cluster were identified using the rank_genes_groups function (sc.tl.rank_genes_groups) with default parameters. A cell cluster was assigned to a specific T-cell subtype or state if there was a substantial overlap (>50%) between its marker genes and the literature markers for that subtype. In addition to the cell type annotation conducted as part of TcEVdb, the dataset has also contributed to the development of TCellAtlas (https://guolab.wchscu.cn/TCellAtlas) and Single T Cell Annotation Tool (STCAT) (https://github.com/GuoBioinfoLab/STCAT). TCellAtlas serves as a reference for T-cell transcriptomic profiles, encompassing 68 subtypes and states derived from 1.6 million T cells, while STCAT is a specialized and automated annotation tool for T-cell subtype and state identification.

### SEVtras-based sEV droplet identification

To identify sEV-containing droplets in scRNA-seq datasets, we employed the SEVtras algorithm, a computational approach specifically designed for detecting EV signals at single-droplet resolution [13]. SEVtras utilizes an expectation-maximization framework, starting with a curated gene set enriched for EV markers, to iteratively compute droplet-specific EV signal scores. Through this process, cell-free droplets are classified as either sEV-containing or not based on their transcriptional profiles. The algorithm quantifies the enrichment of sEV marker genes in each droplet while effectively distinguishing these signals from background noise, such as cellular debris. By aggregating the results across samples, SEVtras generates a unified scoring system that facilitates cross-sample comparisons. This computational method allows for high-throughput identification of sEV populations from large-scale scRNA-seq datasets without requiring additional experimental steps. SEVtras has been validated on multiple datasets, demonstrating its robustness in capturing EV heterogeneity and secretion activity at the single-droplet level.

### EV droplet identification and EV secretion activity index calculation

We employed the SEVtras package to identify EV-containing droplets and calculate EV secretion activity across each dataset [13]. The sEV_recognizer method was employed with default parameters to infer gene expression profiles of EV droplets. It is important to note that while SEVtras identifies droplets containing EVs, these droplets likely contain multiple EVs rather than a single vesicle, as EVs from similar cell types tend to form aggregates [13, 20]. In cases where datasets contained only one sample, we adhered to the SEVtras recommendation of duplicating the data to enhance result reliability. Subsequently, we applied the ESAI_calculator method to calculate EV secretion activity for each sample and each cell type within the samples, utilizing the inferred EV droplet expression profiles alongside the annotated T-cell counts. Default parameters were maintained throughout this calculation.

### EV droplet data processing

Following the identification of EV-containing droplets using SEVtras, projects that yielded fewer than 100 detected droplets were excluded from further analysis. Utilizing the Scanpy, gene filtering was applied to retain only those genes detected in more than three droplets. Data normalization was performed using the function sc.pp.normalize_total with a target sum of 100. Highly variable genes were identified using sc.pp.highly_variable_genes, selecting the top 1000 genes. To mitigate batch effects between samples, sc.external.pp.bbknn was employed. Dimensionality reduction was performed using sc.tl.umap, followed by unsupervised clustering with sc.tl.leiden at a resolution of 0.5. For each cluster, marker genes were identified using sc.tl.rank_genes_groups with the Wilcoxon test. Significant markers were defined as those exhibiting a log fold change >0.5 and an adjusted *P*-value of <0.05. Gene Ontology (GO) and Kyoto Encyclopedia of Genes and Genomes (KEGG) pathway enrichment analyses were performed for the marker genes of each cluster using the clusterProfiler package [21], applying the Benjamini–Hochberg adjustment for multiple testing with a *q*-value cutoff of 0.05.

### Cell atlas–level integration

To integrate single-cell data from multiple projects, we employed scVI for batch correction [22, 23]. The standard Scanpy preprocessing pipeline was also applied, which included normalization, log transformation, and the selection of 2000 highly variable genes using the “seurat_v3” flavor. The scVI model was configured with the following parameters: n_layers = 2, n_latent = 30, and gene_likelihood = “nb”. After training the model, latent representations were extracted and utilized for neighbor graph construction, UMAP visualization, and Leiden clustering (resolution = 0.8).

### Database construction

The TcEVdb database web interface was developed using Vue.js (https://vuejs.org/) and Element Plus (https://element-plus.org) for the frontend, with a Flask (https://flask.palletsprojects.com/en/3.0.x) backend and MongoDB (https://www.mongodb.com/) for data storage. Interactive visualizations were implemented using ECharts (https://echarts.apache.org) and Plotly.js (https://plotly.com/javascript).

## Results

### Database overview and data summary

**Figure 1.**
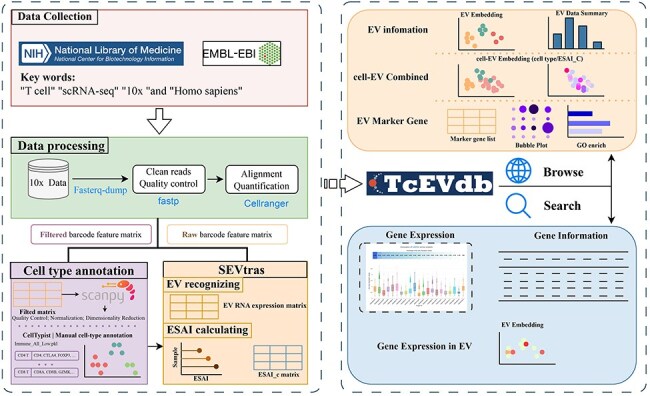
Overview of TcEVdb data collection, analysis workflow, and functionality.

TcEVdb was designed as a user-friendly platform for researchers to explore EV populations across T cells in different tissues and diseases, facilitating the identification of novel EV populations and their potential functions in health and disease. By integrating extensive EV data with detailed metadata and intuitive visualization plots, TcEVdb aimed to accelerate research on TcEV (Fig. 1).

To comprehensive investigate TcEV, we performed EV droplet data processing and obtained 21 scRNA-seq datasets from 221 samples, covering 9 tissue sources and 23 disease states or therapeutic conditions. After analysis, we identified 277 265 EV droplets from 51 T-cell subtypes of these datasets.

**Figure 2.**
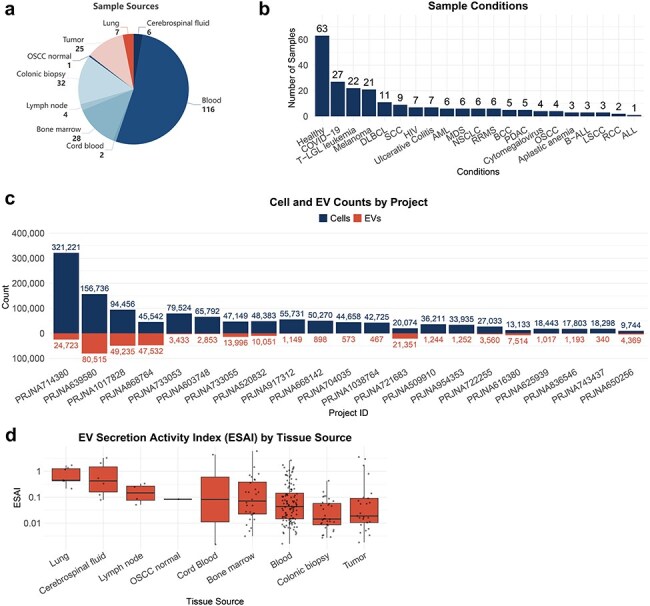
Sample distribution and ESAI across tissue sources of TcEVdb.

TcEVdb encompassed samples from nine tissue sources, with a predominance of blood samples. The analysis of TcEVdb samples showed that blood samples constituted the largest proportion at 52.5% (*n* = 116), followed by colonic biopsies at 14.5% (*n* = 32) and bone marrow at 12.7% (*n* = 28) (Fig. 2a). The disease landscape presented in TcEVdb exhibited considerable diversity, encompassing samples from both healthy individuals and various pathological conditions (Fig. 2b). Notably, healthy samples constituted the largest subgroup (28.5%, *n* = 63). Among the pathological conditions, Coronavirus Disease 2019 (12.2%, *n* = 27), T-cell large granular lymphocytic leukemia (T-LGL leukemia) (10.0%, *n* = 22), and melanoma (9.5%, *n* = 21) were the most prevalent. To illustrate the data scale in TcEVdb, we presented the cell and EV counts for each project (Fig. 2c). The sizes of these projects varied widely, with the largest project containing 321 221 cells and 24 723 EV droplets (PRJNA714380 [24]), while the smallest project contained 9744 cells and 4369 EV droplets (PRJNA650256 [25]).

One of the key features of TcEVdb was the inclusion of EV secretory activity index data. Figure 2d illustrates the distribution of EV secretion activity index (ESAI) values across various tissue sources, demonstrating significant differences in EV secretory activity among these tissues. Notably, the highest median ESAI value was observed in lung-derived samples, followed closely by cerebrospinal fluid and lymph node samples. This finding suggested a potential tissue-specific regulation of EV secretion by T cells, which may be crucial for understanding the role of EVs in local immune responses and tissue homeostasis.

### Database functions and features

**Figure 3.**
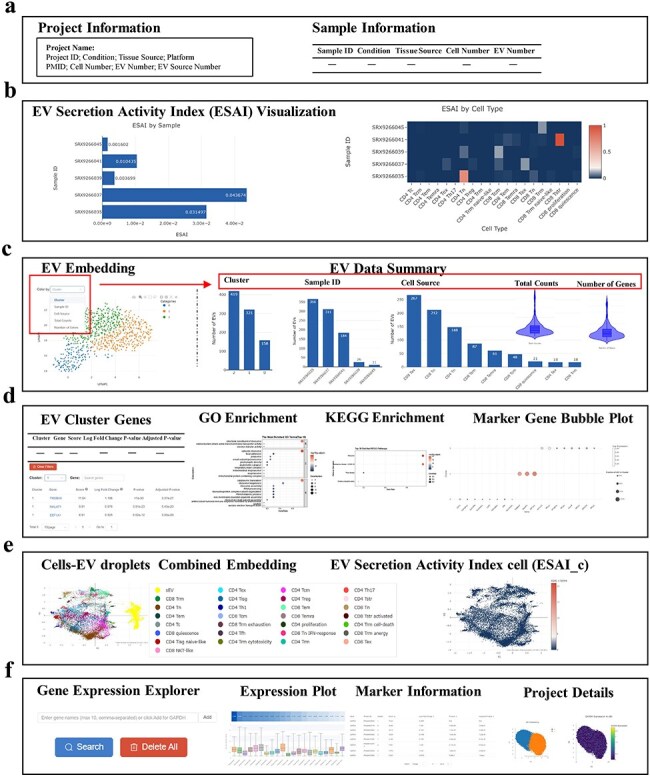
Key features and functions of TcEVdb.

TcEVdb provided a user-friendly interface with two main functions: data browsing and gene querying, providing researchers to investigate the complex landscape of TcEV across biological conditions. The project overview interface (Fig. 3a) offered users comprehensive information about each project, displaying essential metadata such as project name, conditions, tissue sources, and sequencing platforms. The project information panel summarized key metrics, including cell and EV numbers, while the sample information table provided a detailed view of individual samples.

A key feature of TcEVdb was its visualization of ESAI (Fig. 3b). TcEVdb employed interactive heatmaps and bar charts to illustrate ESAI patterns at both the sample and cell type levels, providing valuable insights into the heterogeneity of EV production within a sample. The visualization capabilities of TcEVdb extended to EV droplet analyses through interactive UMAP plots, allowing users to explore EV distribution based on various metadata categories (Fig. 3c). Complementary statistics, such as cluster composition, sample distribution, and cell type origin, provided a multifaceted view of EV subpopulation heterogeneity. Furthermore, gene expression patterns could be visualized via violin plots, facilitating the identification of EV subpopulations with distinct molecular profiles. TcEVdb also provided detailed analyses for each cluster of EVs, including information on DEGs and their functional enrichment for each cluster (Fig. 3d). Users could query DEGs, GO terms, and KEGG pathway lists and images associated with each cluster. These functionalities facilitated the identification of potential functions of specific EV subpopulations.

Another notable attribute of TcEVdb was the integrated visualization of cells and EV droplets (Fig. 3e), facilitating a direct comparison between cellular transcriptomes and those of their secreted EV. The ESAI value for each cell was visually represented in the cell type–level ESAI (ESAI_c) Embedding section. Additionally, the TcEVdb query function (Fig. 3f) enabled users to search for specific genes of interest across all datasets. Users could visualize the expression distribution of a given gene across different projects, identify datasets where that gene served as a cluster marker, and examine its expression pattern across datasets.

### Analysis of T-cell-derived EV from blood samples

**Figure 4.**
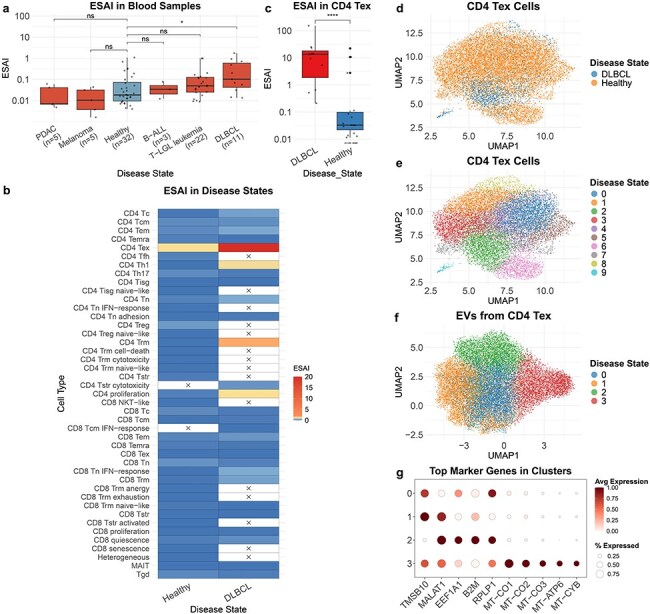
Analysis of TcEV from blood samples.

TcEVdb contains EV from T cells in blood samples across a diverse range of disease states, demonstrating its potential to reveal disease-specific EV profiles. A comparative analysis of ESAI in blood samples from different cancer conditions showed significant differences (Fig. 4a). Notably, ESAI was significantly elevated in diffuse large B-cell lymphoma (DLBCL) compared to healthy controls (*P* ≤ .05). Further investigation into cell type–specific EV secretion patterns in DLBCL and healthy samples revealed different characteristics of various T-cell subsets (Fig. 4b). Among these, CD4^+^ T exhausted cells (CD4 Tex) in DLBCL samples exhibited significantly high ESAI. To validate this, we performed a comparison of CD4 Tex-derived EV secretion between DLBCL and healthy samples (Fig. 4c). The analysis confirmed that ESAI was significantly increased in DLBCL CD4 Tex cells (*P* ≤ .0001), suggesting a potential role of these cells in the pathogenesis or progression of DLBCL.

To elucidate the underlying cellular heterogeneity, we performed cell atlas–level integration of single-cell transcriptomic data of CD4 Tex cells from DLBCL and healthy samples using scVI (Methods: Cell atlas–level integration) (Fig. 4d and e). The integrated analysis encompassed 14 243 cells, with 12 719 cells from healthy samples and 1524 cells from DLBCL samples. Although distinct disease-associated clusters were not immediately discernible, DLBCL-derived cells exhibited a tendency to cluster within Cluster 2 (Fig. 4d). This observation indicates potential transcriptional differences in CD4 Tex cells between DLBCL and healthy samples.

To further characterize DLBCL CD4 Tex cell-derived EVs, we conducted a downscaling and clustering analysis of the EV transcriptome data (Fig. 4f). The results revealed four distinct EV clusters, each characterized by unique gene expression features (Fig. 4g, Supplementary Table S3). Cluster 1 exhibited high expression levels of TMSB10 and MALAT1, genes associated with cell motility and metastasis [26, 27]. Cluster 2 demonstrated elevated expression of EEF1A1, B2M, and RPLP1. Cluster 3 was primarily defined by mitochondrial genes (MT-CO1, MT-CO2, MT-CO3, MT-ATP6, and MT-CYB), suggesting the presence of a distinct EV subpopulation enriched in mitochondrial cargos.

## Discussion

Recent studies have increasingly recognized the importance of T-cell-derived EVs (TcEV) in modulating immune responses and influencing disease progression [28, 29]. Despite the increasing acknowledgment of their significance, the investigation of TcEV has been hindered by the lack of comprehensive resources for analyzing their heterogeneity. To address this gap, we developed TcEVdb, a database that integrated large-scale single-cell RNA sequencing data processed using the SEVtras algorithm, thereby providing unprecedented insights into TcEV. TcEVdb offered a comprehensive platform for the exploration of TcEV across various biological conditions.

Several databases have been established to enhance research on EVs, but TcEVdb introduces a novel approach utilizing scRNA-seq data, which enables droplet resolution identification of sEV. TcEVdb focuses exclusively on T-cell-derived EVs, providing detailed characterization across 51 T-cell subtypes. Unlike Vesiclepedia 2024 and exoRBase 2.0 [30, 31], which aggregate data across diverse cell types without resolving cell-specific dynamics, TcEVdb’s specificity facilitates the exploration of the heterogeneity and secretion activity of T-cell EVs at single-cell resolution. Additionally, ExoBCD focuses primarily on biomarkers for breast cancer [32], and EVAtlas and EVmiRNA enrich EV research by concentrating on microRNA expression profiles and their functional implications [33, 34]. However, these resources are limited to bulk-level datasets. By employing scRNA-seq datasets, TcEVdb not only uncovers EV secretion patterns but also provides cell-specific biological insights, establishing it as a powerful resource for advancing our understanding of T-cell-derived EVs.

The development of TcEVdb involved a comprehensive process of data collection, processing, and analysis. T-cell scRNA-seq datasets were collected from public databases and underwent rigorous quality control and preprocessing. Detailed cell type annotation was accomplished through a combination of automated and manual methods. Subsequently, the SEVtras algorithm was applied to identify EV-containing droplets and calculate EV secretion activity, yielding insights into EV heterogeneity and secretion patterns.

However, certain limitations of our study should be acknowledged. The droplets identified by SEVtras likely contain multiple EVs rather than individual vesicles, due to the propensity of EVs from similar cell types to aggregate [20]. This aggregation, while potentially enhancing our ability to detect EV-related transcripts, limits our resolution to clusters of EVs rather than individual vesicles. As a result, the multi-EV nature of our droplets results in a higher number of detected genes per droplet compared to the recent single-EV study [12]. Additionally, the current version of TcEVdb focuses exclusively on TcEV, which restricts our understanding of the broader EV landscape. The biological significance of EVs extends beyond TcEV and immune regulation, with EVs from numerous cell types contributing to diverse physiological processes [35, 36]. Future iterations of the database could benefit from including EV-containing droplets derived from other immune and nonimmune cell types, providing a more comprehensive view of intercellular communication networks in various physiological and pathological contexts.

The case study of TcEV from blood samples exemplified the potential of TcEVdb in generating novel biological insights. Our results revealed a disease-specific pattern of EV secretion, particularly in DLBCL. The observed increase in EV secretion from CD4 Tex cells in DLBCL, along with the characterization of various EV subpopulations, may provide a foundation for future studies investigating the role of TcEV in the pathogenesis of cancers [37].

In summary, TcEVdb was the first dedicated database for EVs derived from T cells, offering a platform for exploring the EV transcriptome across various biological conditions. TcEVdb included interactive visualization, gene expression profiling, and multigene search capabilities, enabling researchers to conduct comprehensive functional analyses of EV subpopulations. As the field of EV research continues to evolve, TcEVdb is anticipated to advance EV biology research and contribute to the development of innovative EV-based diagnostics and therapeutics for a diverse array of diseases.

## Supplementary Material

baaf012_Supp

## Data Availability

All raw data used in this study were obtained from publicly available databases. The accession numbers used in this study are provided in Supplementary Table S1. Additionally, all processed data, metadata, are freely accessible through TcEVdb (https://guolab.wchscu.cn/TcEVdb).
